# Basal Ganglia Neuronal Activity during Scanning Eye Movements in Parkinson’s Disease

**DOI:** 10.1371/journal.pone.0078581

**Published:** 2013-11-06

**Authors:** Tomáš Sieger, Cecilia Bonnet, Tereza Serranová, Jiří Wild, Daniel Novák, Filip Růžička, Dušan Urgošík, Evžen Růžička, Bertrand Gaymard, Robert Jech

**Affiliations:** 1 Department of Neurology and Center of Clinical Neuroscience, Charles University in Prague, 1st Faculty of Medicine and General University Hospital, Prague, Czech Republic; 2 Department of Cybernetics, Faculty of Electrical Engineering, Czech Technical University, Prague, Czech Republic; 3 Department of Stereotactic and Radiation Neurosurgery, Na Homolce Hospital, Prague, Czech Republic; 4 CRICM UPMC/INSERM UMR_S975, CNRS UMR7225, ICM, Pitié-Salpêtrière Hospital, Paris, France; 5 Pierre et Marie Curie Paris-6 University, Paris, France; Beijing Institute of Radiation Medicine, China

## Abstract

The oculomotor role of the basal ganglia has been supported by extensive evidence, although their role in scanning eye movements is poorly understood. Nineteen Parkinsońs disease patients, which underwent implantation of deep brain stimulation electrodes, were investigated with simultaneous intraoperative microelectrode recordings and single channel electrooculography in a scanning eye movement task by viewing a series of colored pictures selected from the International Affective Picture System. Four patients additionally underwent a visually guided saccade task. Microelectrode recordings were analyzed selectively from the subthalamic nucleus, substantia nigra pars reticulata and from the globus pallidus by the WaveClus program which allowed for detection and sorting of individual neurons. The relationship between neuronal firing rate and eye movements was studied by crosscorrelation analysis. Out of 183 neurons that were detected, 130 were found in the subthalamic nucleus, 30 in the substantia nigra and 23 in the globus pallidus. Twenty percent of the neurons in each of these structures showed eye movement-related activity. Neurons related to scanning eye movements were mostly unrelated to the visually guided saccades. We conclude that a relatively large number of basal ganglia neurons are involved in eye motion control. Surprisingly, neurons related to scanning eye movements differed from neurons activated during saccades suggesting functional specialization and segregation of both systems for eye movement control.

## Introduction

In everyday life we scan the environment with a series of eye movements, pointing the fovea towards objects of interest and the most salient areas of the scene. The pattern of such eye movements (EM) carried out while exploring an image, also called scanning EM, is composed of a succession of small saccades and fixations, corresponding to successive re-allocation of attention from one detail to another [1], [2]. Therefore, scanning EM can be considered as internally triggered EM, as the subject moves the gaze around a complex visual image actively searching for information relevant to current motivations and goals. The visual scanpath is generated by complex parallel strategies [3] and depends on planning, visuospatial attention, spatial working memory and emotional state [4], [5]. Scanning EM have mostly been the domain of psychiatric research which has focused on the behavioral aspects of the eye scanning path rather than to pathophysiological origin and scanning EM control [6].

The structures and mechanisms involved in scanning EM are still poorly understood. At the subcortical level, an involvement of the basal ganglia during scanning EM was suggested by early research using regional cerebral blood flow in healthy controls and schizophrenic patients [7]. The importance of the basal ganglia in EM control was further confirmed by animal studies [8], [9], [10], [11], [12], which discovered neurons co-activated during EM by single cell recordings in several regions of the basal ganglia and brainstem [9], [11], [13]. However, subcortical neuronal activity during scanning EM is still unknown and has never been studied in animals or in humans before. Several human studies supported the participation of the basal ganglia in EM control but just with results based on reflexive and voluntary saccades analyzed from oculographic recordings [14], [15], [16], [17], [18], [19] or local field potentials [20]. The only evidence of human EM-related neurons was obtained from the subthalamic nucleus during saccade tasks and smooth pursuit movements in patients with Parkinson’s disease [21].

In our study, we systematically searched for basal ganglia neurons participating in scanning EM. We took advantage of intraoperative microelectrode recordings of single neuronal activity routinely used to identify the basal ganglia based on specific electrophysiological pattern [22]. We have focused on the subthalamic nucleus (STN), substantia nigra pars reticulata (SNr) and globus pallidus (GP) – i.e. nuclei in which EM-related activity was previously reported [11], [13] and which are easily accessible during the implantation procedure for deep brain stimulation in Parkinsońs disease (PD).

Besides EM-related neurons firing selectively when a specific position, velocity or acceleration of the eyeballs is reached, we expected to find less specialized neurons with activity depending on two or more kinematic features simultaneously. This comes from the hypothesis of functional overlap based on neuronal convergence along the striato-pallido-thalamic projection and assuming compression of information when travelling from larger to smaller nuclei [23]. Findings of STN neurons showing co-activation during various eye movement tasks are in agreement with this theory [9], [21]. On the other hand, there is a segregation hypothesis which expects different neuronal populations to selectively respond to specific kinematic parameters or to fire only during a specific kind of the EM. Indeed, functional and anatomical segregation between various EM tasks has been previously observed at different levels involving the cortex, basal ganglia or cerebellum [4], [8], [24]. Therefore, in a subgroup of patients, we additionally studied the basal ganglia neurons during externally triggered EM using a visually guided saccade task. To further elucidate the function of neurons related to EM, we explored temporal relations of EM kinematic parameters with respect to their preceding and following activity, which may suggest their involvement in execution or control processes.

## Methods

### Ethics statement

The study was approved by the Ethics Committee of the General University Hospital in Prague, Czech Republic and was conducted according to the Declaration of Helsinki.

### Patients

Nineteen PD patients were enrolled consecutively from 2008 to 2011 (15 men, 4 women; mean age: 54.5, SD 9.8, range 28–69 years; mean PD duration: 13.8, SD 6.1, range 3–30 years; Hoehn-Yahr stage 2-4; mean motor score of the Unified Parkinsońs Disease Rating scale – UPDRS III in OFF condition: mean 36.5, SD 13.6, range 10–65). All of them were suffering from motor fluctuations and/or disabling dyskinesias (demographic details in Table 1) and were indicated for treatment with deep brain stimulation due to motor fluctuations and dyskinesias. All of them met the UK Brain Bank Criteria for diagnosis of PD [25] and all gave their written informed consent for participation. Patients with dementia and/or depression had been excluded by a routine psychiatric examination and neuropsychological testing (Mini-mental state examination, Mattis dementia rating scale, Beck depression inventory). As a normal cognitive state was requested to fulfill the general indication criteria for implantation surgery, all patients understood the nature of the experiment. They had been informed that procedures related exclusively for study purposes could be skipped if desired. It had been emphasized that they were allowed to forego the experiment at any time before or during the surgery. Four days before surgery, dopamine agonists were substituted by equivalent doses of levodopa. Other anti-PD medication (amantadine, anticholinergics) was suspended earlier for the surgery preparation. Levodopa was withdrawn at least 12 hours before the surgery.

**Table 1:** Description of patients with Parkinson’s disease.

patient	Age [years]	DD [years]	levodopa [mg]	UPDRS III	H-Y	DBS target	task	neurons
1	64	14	1375	31	2.0	STN	SEM	12
2	61	14	1200	37	2.5	STN	SEM	7
3	46	15	1000	40	3.0	STN	SEM	15
4	63	30	1250	50	3.0	STN	SEM	3
5	53	12	700	37	2.5	STN	SEM	14
6	69	9	750	47	3.0	STN	SEM	5
7	49	12	1550	65	4.0	STN	SEM	7
8	59	12	600	30	2.5	STN	SEM	8
9	63	14	1350	21	2.0	GPi	SEM	4
10	53	10	750	42	4.0	GPi	SEM	11
11	53	11	1663	45	2.5	STN	SEM	5
12	57	26	2000	59	4.0	STN	SEM	12
13	28	3	720	10	2.0	GPi	SEM	6
14	64	17	1500	31	2.5	STN	SEM	12
15	53	12	1000	40	4.0	STN	SEM	9
16	44	10	1130	23	2.0	GPi	SEM, VGS	2
17	42	9	740	33	3.0	STN	SEM, VGS	20
18	55	19	1980	35	2.0	STN	SEM, VGS	16
19	60	14	1060	18	2.0	STN	SEM, VGS	15

Age – age on the day of surgery; STN – subthalamic nucleus; GPi – globus pallidus interna; DD – Parkinsońs disease duration; Levodopa – dose/day in mg including levodopa equivalent dosage of dopamine agonist; patient 4 was also treated with mianserin; patients 6, 7, 8, 9, 10 with citalopram and 16 with bupropion; UPDRS III – motor score of the Unified Parkinsońs Disease Rating Scale in OFF medication condition; H-Y – Hoehn and Yahr stage in OFF medication condition; DBS target – nucleus chosen for bilateral deep brain stimulation; SEM – scanning eye movement task; VGS – visually guided saccade task; neurons – number of neurons identified in the basal ganglia.

### Surgery and intraoperative microrecording

Implantation of the deep brain stimulation system was performed separately in two steps: (i) stereotactic insertion of the permanent quadripolar electrode into the STN bilaterally and (ii) implantation of connection leads and the neurostimulator to the subclavial region. The Leksell frame and SurgiPlan software system (Elekta, Stockholm, Sweden) were employed in the stereotactic procedure. Pre-surgical planning was based on 1.5 T MRI with direct visualization of the target. The central trajectory was intentionally focused on the STN center near the anterior part of the red nucleus (15 patients) or to the posteroventrolateral portion of the GP interna (4 patients). The first surgery was performed while awake under local anesthesia. The extracellular neuronal activity was mapped by conventional microelectrode recordings (MER) using parallel insertion of five tungsten microelectrodes spaced 2 mm apart in a “Ben-gun” configuration to select sites for the macroelectrode intraoperative stimulation [26], [27]. Four out of five channels of the Leadpoint recording system (Medtronic, MN) were used for the MER, filtered with 500 Hz high pass filter and 5 kHz low pass filter, sampled at 24 kHz and stored for off-line processing. As the firing pattern of the external globus pallidus could not always be distinguished from the internal globus pallidus, we classified both areas as one structure – GP. Up to six recording positions in the STN, SNr or GP were used for the EM tasks in each patient. The number of positions depended on the time course of the surgery, patients' clinical conditions and compliance. Tasks were not performed if patients demonstrated discomfort from being in the supine position or exhibited painful symptoms relating to the off-medication state as well as increased fatigue or sleepiness during surgery. Immediately after the procedure, the position of each permanent electrode was verified by two orthogonal X-ray images co-registered with a presurgical MRI plan. No dislocation larger than 1 mm was found in any patient.

### Eye movement recording

Eye movements during scanning and visually guided EM tasks were recorded using electrooculography (EOG), a technique measuring the position of the eye in terms of the electric potential induced by the eye dipole. Technical constraints during surgery (limited space around the stereotactic frame and a limited number of recording channels) did not allow for more elaborate recordings than the use of one single-channel EOG. The signal was band-pass filtered in the range of 0.1–20 Hz and recorded using the Leadpoint recording system simultaneously with MER acquisition through a pair of surface electrodes attached near the outer canthus and the lower lid of the left eye. This setup enabled the orthogonal projection of the eye position on the axis connecting the two EOG electrodes. All eye movements except those which were orthogonal to the axis could be recorded with this technique.

### Tasks

The EM tasks were presented on a 17“-computer screen placed approximately 55 cm in front of the eyes of patients lying in supine position.

**Figure 1:**
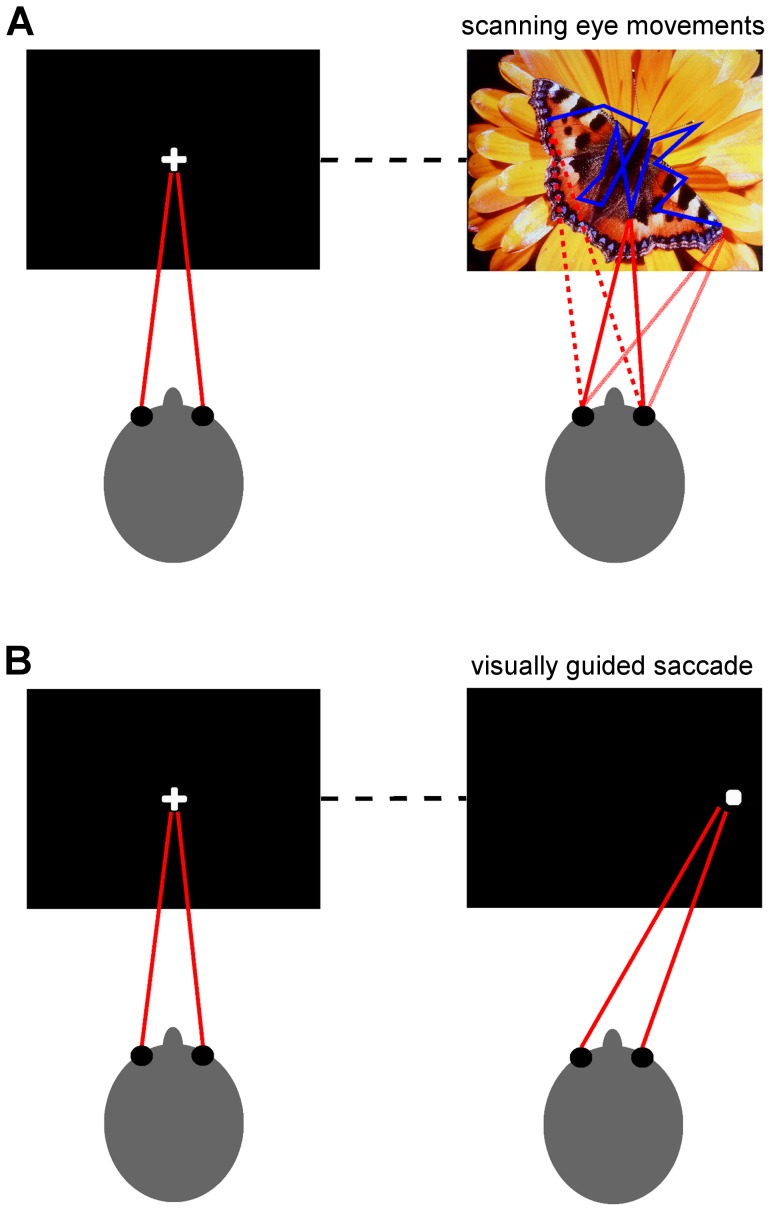
Eye movement (EM) tasks employed in the study. **A - The scanning EM task**. After the presentation of the black screen with a central cross, a photograph chosen from the International Affective Picture System was presented for 2 s. Patients were asked to initially fix their eyes on the cross (left picture) and then simply watch the photograph (right picture). In total, 24 pictures were consecutively used during the task. The blue line highlights a possible eye scanpath. **B - The visually guided saccade task** consisted of a presentation of 10 pairs of indifferent central (left picture) and lateral GO (right picture) targets positioned pseudorandomly on the left/right side of the screen. Patients were instructed to initially fixate the central cross and then track to the lateral targets as fast as possible.

**The scanning EM task.** The goal of this task was to induce self-initiated free-direction scanning EM. The task consisted of a presentation of a series of photographs selected from the International Affective Picture System (IAPS, Figure 1A) [28], depicting objects, persons, animals and landscapes. To avoid showing the same picture more than once, six unique variants of the test, each containing 24 pictures, were prepared. Each picture was presented for a period of 2 s and was preceded by a black screen for various durations (3500–5500 ms) with a white cross in the center. Patients were asked to fix their eyes on the cross on the black screen and then to simply watch the pictures presented. The MER and EOG signals were acquired in 2 s epoch intervals recorded both during the picture presentation and the black screen. The task lasted approximately for 2.5 minutes.

**The visually guided saccade task.** The goal of the task was to induce externally generated horizontal saccades (Figure 1B). Initially, a black screen with a central white cross was shown for a pseudorandom period of 2, 2.25, or 2.5 seconds. Subsequently, a peripheral target, a small white square, was presented for 1 s, 17 degrees laterally from the central fixation cross, pseudorandomly to the left (5 trials) or right (5 trials). Patients were instructed to initially fixate on the central cross and then to track the lateral target as fast as possible. The MER and EOG signals of 2 s durations were recorded during all 10 trials. The task lasted for 32.5 seconds.

### Data analysis

**Figure 2:**
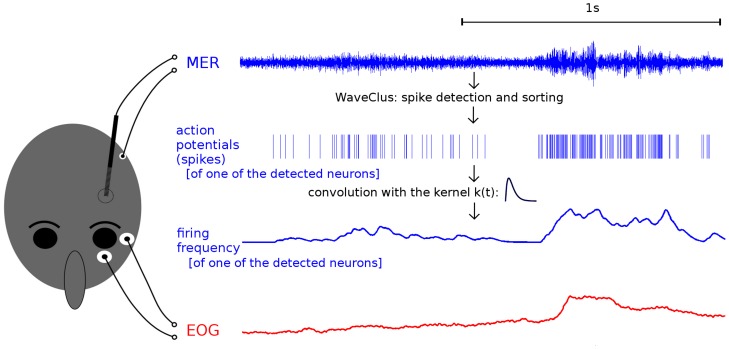
Microelectrode recording (MER) and electrooculography (EOG) signal acquisition and processing. Action potentials of individual neurons were identified using the WaveClus algorithm in the MER signal. The instantaneous firing rate (IFR) was then estimated by convolving a series of extracted action potentials generated by a single neuron with a causal kernel function. Finally, the IFR was correlated with the eye movement kinematic parameters derived from the EOG.

**Microelectrode recordings.** WaveClus [29], an unsupervised spike detection and sorting tool, which performed reasonably well on the single channel MER [30], was used to extract the series of action potentials of individual neurons from MER signals (Figure 2). Instantaneous firing rate (IFR) of each neuron was estimated by convolving the series of action potentials with the causal kernel function α^2^*t*exp(-α*t) defined for positive time *t*, where 1/α was empirically set to 20 ms.

Each neuron was then mapped relative to the border of the STN, GPi and SNr identified by intraoperative MER. One-dimensional positions along the dorso-ventral microelectrode trajectory were determined using this technique (Figure S1).

**EM recordings.** EOG signals were rated manually and those contaminated with technical or major blinking artifacts, usually represented by large amplitude changes oversaturating the recording channel, were excluded from further analyses. As we presumed that neuronal activity could be related not only to the position of the eye, but also to its motion and the dynamics of the motion [21], [31] we characterized EM by: i) the eye position (POS), defined by the EOG signal itself, ii) the eye velocity (VELOC), defined as the derivative of POS, and iii) the acceleration of the eye (ACCEL), defined as the derivative of VELOC. The derivative of the signal was defined in terms of the differences between successive samples in a low-pass filtered signal computed using a sliding rectangular window with the cutoff frequency of 12.5 Hz. The maximum and typical amplitude of the EM was extracted in each recording position in each task for each patient. While the maximum amplitude was defined as the extreme value in VELOC, the typical amplitude was defined as the median peak exceeding ±1 SD of the VELOC.

**Figure 3:**
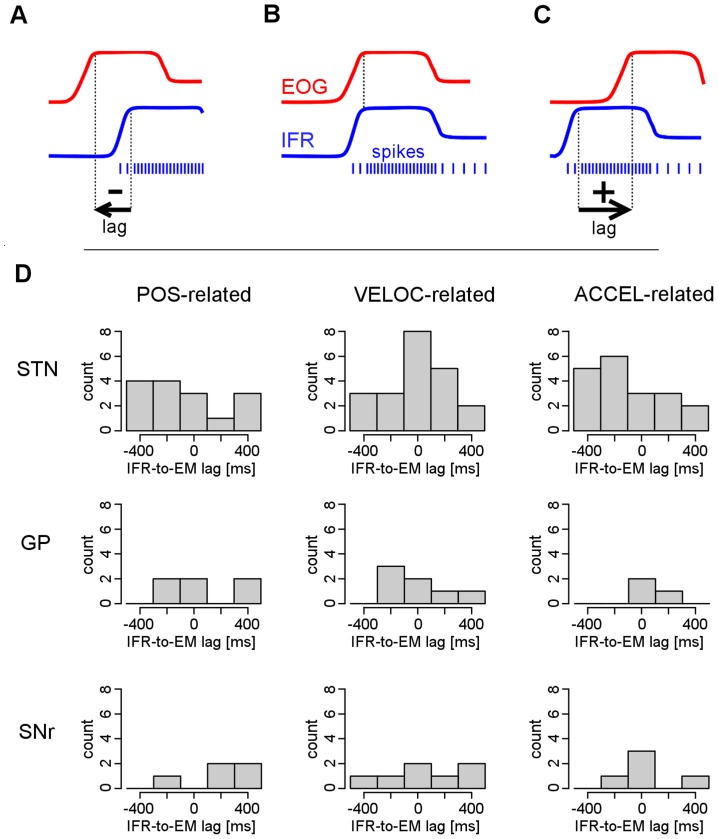
Time lag of neuronal activity with respect to electrooculography (EOG). A, B, C - Explanation of the cross-correlation procedure in three examples. Action potentials of three hypothetical neurons along with corresponding instantaneous firing rate (IFR) were correlated with the theoretical EOG signal. Figure A – the IFR correlates with the past EOG signal suggesting a sensory function of the neuron. Figure B – the IFR correlates with the concurrent EOG signal suggesting an executive function of the neuron. Figure C – the IFR correlates with the future EOG signal suggesting a preparatory function of the neuron. The time lag of the IFR in which the maximal (and significant) correlation with EM is reached is called the *optimal IFR-to-EM cross-correlation lag*. This lag is negative in A, zero in B and positive in C. Figure D - Frequency histograms of the optimal instantaneous firing rate (IFR) to eye movement cross-correlation lags in all eye movement-related neurons during the scanning eye movement task across the subthalamic nucleus (STN), globus pallidus (GP), and substantia nigra pars reticulata (SNr) considering kinematic parameters of the electrooculography (POS, VELOC, ACCEL, in columns). No significant differences in the locations of these distributions were found.

To identify neurons whose activity was associated with EM, the relationships between IFR and POS, IFR and VELOC, and IFR and ACCEL were assessed. A neuron was considered connected to EM if its IFR was related to at least one of POS, VELOC, and ACCEL at the Bonferroni-corrected significance level of *p*<0.05. The relationships between IFR and the EM characteristics were analyzed using cross-correlation, which could reveal not only the link between concurrent IFR and EM, but also the link of IFR to preceding and following EM (Figure 3A-C). The maximal cross-correlation lag considered was ±500 ms with steps of 2.5 ms. Biased estimates of correlation coefficients were computed to diminish uncertainty in estimates of correlation coefficients over longer lags. The cross-correlation coefficient between two signals was defined as the extreme correlation coefficient between the signals over all the lags considered. The lag in which the extreme cross-correlation was reached was called the *optimal EM-to-IFR cross-correlation lag*. The statistical significance of the cross-correlation coefficient between two signals was assessed with Monte-Carlo simulations [32], [33] using original and surrogate signals generated by randomly changing the phases of the spectral representation of the original signal.

The binomial test, Pearson's correlation coefficient test, Fisher exact test, two-sample proportion test, likelihood ratio test comparing Poisson regression models of dependence and independence in a 2-by-2-by-2 contingency table and paired t-test were used for statistical analysis. Data processing and analyses were performed in MATLAB (R2007b, The MathWorks, Natick, MA) and “R” software [34].

## Results

We acquired 137 pairs of MER and EOG signals from 91 recording positions: 97 MERs were assigned to the STN, 21 to the GP and 19 to the SNr according to their firing pattern. In total, 183 neurons were detected using the spike sorting procedure, out of which 130 were located in the STN, 23 in the GP and 30 in the SNr (Table 2).

**Table:** Table 2. Numbers of microelectrode recordings and neurons detected.

	STN	GP	SNr	Total
MER count	97	21	19	137
neuron count (SEM task)	130	23	30	183
neuron count (SEM & VGS task)	46	2	5	53

MER count – number of microelectrode recordings obtained in each nucleus; SEM – scanning eye movement task; VGS – visually guided saccade task; neuron count – number of neurons identified in each nucleus during the SEM task (patients 1-19) and during both the SEM and VGS tasks (patients 16-19); STN – subthalamic nucleus; GP – globus pallidus; SNr – subtantia nigra pars reticulata.

### Neuronal activity related to scanning eye movements

Thirty seven (20%) out of 183 neurons identified in the basal ganglia during the scanning EM task were related to at least one of the EM kinematic parameters (POS, VELOC, ACCEL) (Table 3). Their proportion was higher than the expected false positive rate in each of the analyzed nuclei (binomial test, *p*<0.001): 26/130 neurons (20%) in the STN, 5/23 neurons (22%) in the GP and 6/30 neurons (20%) in the SNr. Locations of the EM-related neurons are depicted in the Figure S1. In the STN, the ratio of the EM-related neurons was higher in the ventral part (0 to 1 mm from the ventral STN border) compared to the rest of the nucleus (proportion test, χ^2^ = 2.722, df = 1, P<0.05).

**Table 3:** Number of neurons related to eye movements in the scanning eye movement task.

	STN (130 neurons)	GP (23 neurons)	SNr (30 neurons)	Total (183 neurons)
EM-related neurons^†^	26 (20%)***	5 (22%)***	6 (20%)***	37 (20%)***
POS-related	15 (12%)**	6 (26%)***	5 (17%)*	26 (14%)***
VELOC-related	21 (16%)***	7 (30%)***	7 (23%)***	35 (19%)***
ACCEL-related	19 (15%)***	3 (13%)	5 (17%)*	27 (15%)***
POS & VELOC-related	10 (8%)	4 (17%)*	5 (17%)*	19 (10%)**
POS & ACCEL-related	7 (5%)	3 (13%)	3 (10%)	13 (7%)
VELOC & ACCEL-related	10 (8%)	3 (13%)	4 (13%)	17 (9%)*
POS & VELOC & ACCEL-related	7 (5%)	3 (13%)	3 (10%)	13 (7%)

EM-related neurons – the number of eye movement-related neurons associated with at least one kinematic parameter (^†^Bonferroni-corrected number of neurons for three kinematic parameters). Neurons functionally associated with one or more kinematic parameters (POS – eye position; VELOC – eye velocity; ACCEL – eye acceleration) are reported for each nucleus separately (STN – subthalamic nucleus; GP – globus pallidus; SNr – substantia nigra pars reticulata). Number of neurons significantly greater than expected 5% false positivity rate is denoted: *(*p*<0.05), **(*p*<0.01) ***(*p*<0.001).

**Figure 4:**
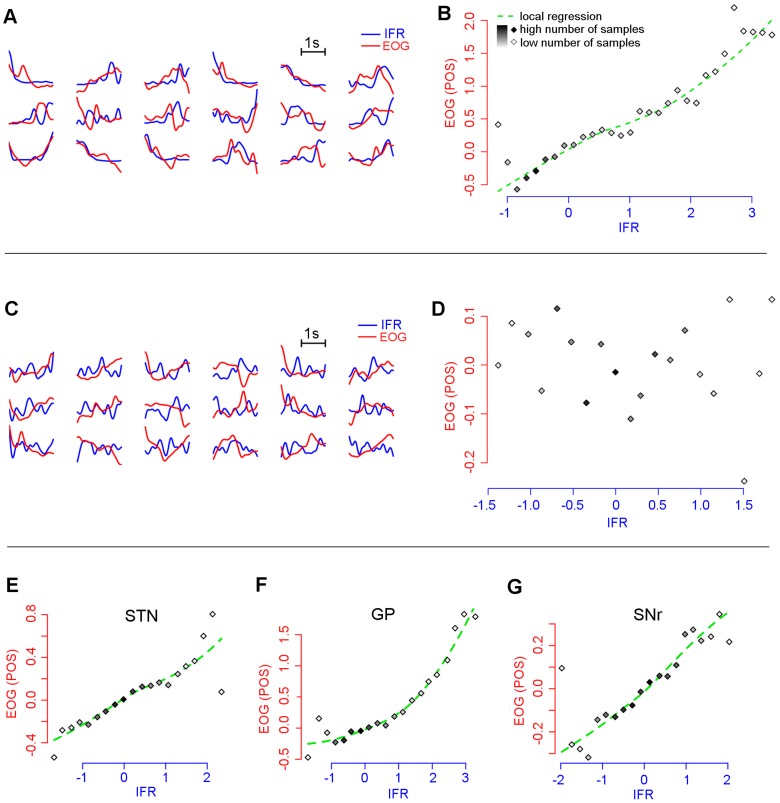
Neuronal activity during the scanning movement task. Example of neuron related (A, B) and unrelated (C, D) to eye movements based on correlation analysis of the instantaneous firing rate (IFR) and eye position (POS) derived from the electrooculography (EOG). All eye movement-related neuronal populations in the STN, GP and SNr are plotted in figures E, F, and G. Figures A, C show the IFR (blue) and EOG (red) pairs recorded during epochs of the task involving both the black screen and pictures presentations. Figures B, D, E, F, G show the dependency of the normalized eye position (POS) derived from the electrooculography (EOG) on the normalized, sorted and binned amplitude of the instantaneous firing rate (IFR). While the IFR from a single neuron was used on figures B and D; the IFR from all eye sensitive neurons were used on figures E, F, and G for each nucleus separately. The amplitudes of the POS signals which correlated negatively with the IFR signal were reversed. The number of signal samples in each bin is expressed by different shades of grey in the diamond glyphs.

The firing rate of the neurons relating to eye position (POS) significantly correlated with fluctuations of the EOG (Pearson’s r = 0.89 (STN), 0.91 (GP), 0.86 (SNr); df = 18, p<0.001) (Figure 4). A relatively large number of neurons were related to more than one kinematic parameter (likelihood ratio test, D = 42.2 (STN), 19.8 (GP), 28.0 (SNr); df = 3, *p*<0.001).

As follows from cross-correlation analysis, the firing rate of the neurons was related either to concurrent, previous, or future EM (Figure 3). However, none of the nuclei predominantly contained any kind of the time-related neurons.

### Neuronal activity related to visually guided saccades

There were 10/46 neurons (22%) whose activity was related to visually guided saccades in the STN, 1/2 of the neurons were in the GP and 2/5 were in the SNr. A description of neurons related to all EM kinematic parameters (POS, VELOC, ACCEL) is shown in Table 4.

**Table 4:** Eye movement-related neurons detected in the scanning eye movement task and/or visual guided saccade tasks.

	STN (46 neurons)			GP (2 neurons)			SNr (5 neurons)		
	SEM	VGS	Both	SEM	VGS	Both	SEM	VGS	Both
**EM-related neurons** ^†^	**10**	**10**	**2**	**0**	**1**	**0**	**1**	**2**	**0**
POS-related	4	9	0	0	0	0	0	2	0
VELOC-related	9	4	1	0	0	0	1	0	0
ACCEL-related	8	11	3	0	1	0	2	0	0
POS & VELOC-related	3	4	0	0	0	0	0	0	0
POS & ACCEL-related	2	4	0	0	0	0	0	0	0
VELOC & ACCEL-related	4	2	0	0	0	0	1	0	0
POS & VELOC & ACCEL-related	2	2	0	0	0	0	0	0	0

EM-related neurons – the number of eye movement-related neurons associated with at least one kinematic parameter (^†^Bonferroni-corrected number of neurons for three kinematic parameters) identified from patients 16-19 which performed both the scanning eye movement task (SEM) and visual guided saccade task (VGS) in the subthalamic nucleus (STN), globus pallidus (GP) and substantia nigra pars reticulata (SNr). Neurons functionally associated with one or more kinematic parameters (POS – eye position; VELOC – eye velocity; ACCEL – eye acceleration) are reported for each nucleus separately.

### Eye movements in the scanning and saccadic tasks

As both the scanning EM and visually guided saccades tasks were executed by only four patients, 19 relevant recording positions were analyzed. Neurons related to scanning EM were usually not activated in the visually guided saccades task and vice versa. Out of 46 STN neurons found in these patients, ten neurons related to scanning EM, ten neurons related to visually guided saccades and only two were activated during both tasks. These neuronal populations seemed to be independent in each of the two tasks as no evidence against the null hypothesis of independence was found (Fisher exact test, *p* = 1.0) although the test had enough power to reject the null hypothesis had the number of co-activated neurons been higher. In the GP and SNr, an insufficient number of neurons were detected for proper assessment of independence in neuronal activity between the two tasks. However, no GP or SNr neurons were co-activated during both tasks.

Descriptive analyses of the EM amplitude revealed that the maximal amplitude of the scanning EM and visually guided saccades were nearly identical. As requested by the visually guided task, patients executed large saccades, while small EM predominated in the scanning task where large EM occurred only rarely. The amplitude of the typical EM made during the visually guided saccades task was greater than during the scanning task (t = 5.7, df = 18, p<0.001). On average, the median saccade amplitude was 2.6 times larger in the visually guided task than in the scanning EM task.

## Discussion

We showed that nuclei of the basal ganglia (namely, STN, GP and SNr) contain neurons whose firing rates correlated with eye movements during the scanning EM task. The proportion of EM-related neurons was relatively high reaching 20-22% in each of those nuclei (Table 3). Despite technical limitations due to the single-channel EOG recording we found relationships between different kinematic parameters of the EM and the firing rate in many neurons (Table 3, Figure 4). These findings point to the role of the basal ganglia in the static and dynamic representation of the EM, a role of importance for the maintenance of accuracy in goal-directed movements.

### Eye movement activity in basal ganglia

Our single unit records from the STN showed that the proportion of EM-related neurons was higher in its ventral part (Figure S1). A 20% share of oculomotor neurons in the ventral part of the STN has already been noted in monkeys [10] and in humans [21]. However, those were solely neurons involved in saccadic EM. As suggested by our results, the SNr and GP are probably as equally important for control of voluntary scanning EM as the STN. We consider this as one of the major outcomes of our study because in both of these nuclei, the oculomotor activity had previously been noted during EM only in animals [11], [13], [35].

The role of the STN in EM has been largely explored in deep brain stimulation treated patients with Parkinson’s disease. A high intensity STN neurostimulation resulted in contraversive eyeball deviations [36], [37], similar to STN inactivation after locally injected GABA in animals [38] or after unilateral traumatic striato-subthalamic lesion [39]. An electrode penetration to the STN has an impact on the EM parameters as well. It causes a transitory microlesion [40] prolonging the latency of reflexive saccades [41] which are already prolonged due to Parkinson’s disease [42]. Unlike microlesion, deep brain stimulation has an opposite effect on the STN as the latency of visually initiated reflexive saccades become shorter and normalized [41], [43], [44] while their gain is growing [45]. In addition, the STN deep brain stimulation improves some of the parameters of voluntary saccades [18], [46] and suppresses interruptive saccades during fixation [47].

The significance of the STN in EM control is also well documented by other studies. The STN participates in the initiation of voluntary EM and in the inhibition of automatic EM [9], [46], probably reflecting the influence of a hyperdirect pathway connecting the SMA and the motor cortex [48], [49], [50] including the supplementary eye field [51] with the STN, bypassing slower projections through the basal ganglia [52], [53]. By the hyperdirect pathway, the motor plan can be rapidly implemented at the STN level and interfere with automatic EM [9], [11]. The STN influence is then propagated by the following two main outputs [54], [55]. The first is an excitatory glutamatergic projection to the SNr [56], whose activity is reduced or increased during saccades or smooth pursuit movements [12], [13], [35]. The SNr subsequently sends ipsi- as well as contralateral projections to the superior colliculus [57] which is an important nucleus involved in the control of automatic reflexive saccades [8]. The second glutamatergic output from the STN projects to the internal part of the GP [56] through which the oculomotor pattern can be further modified. The GP is more than just a skeletomotor structure as confirmed by several findings of EM-related neurons in its external and internal role during visually guided saccades [11] and anti-saccades in animals [58]. Moreover, bilateral pallidotomy affects the fixation [17] and reduces the velocity of self-initiated saccades [15]. On the other hand, deep brain stimulation of the GP interna modifies other parameters of automatic as well as voluntary saccades (Fawcett et al., 2005). Hence the fact that during the scanning EM task we found EM-related neurons in the STN, SNr and GP was not surprising.

### Segregation and convergence in eye movement control

Scanning EM are an important tool in the exploration of complex visual stimuli [6], [59]. Their trajectory is made up of a sequence of variably large saccades and fixations with the visual field maintained for tens to hundreds of milliseconds. As a result, a certain detail is steadily projected on the fovea. This is followed by a saccade, a rapid voluntary movement, by means of which the fovea moves on to a new point of interest while information from the other parts of the retina is being concurrently assessed in search of another point of fixation. This distributed parallel processing has been recently confirmed by the sequential scanning task [60]. As expected, in four patients where both tasks were used, the median amplitude of scanning EM was smaller than that of the saccades in the visually guided task. At the same time, the amplitudes of largest EM executed in both tasks were similar. This is in agreement with previous studies, indicating that the amplitudes of scanning EM follow a heavily skewed distribution towards low values, with relatively rare movements of larger amplitude [61].

From what structures and in which way the scanning movements are controlled is still poorly understood. Since they are under voluntary control, they can be seen as a model with internally generated movements – unlike reflexive saccades which are initiated by external stimuli. Internally and externally triggered movements are generally subject to different control and executive mechanisms [62], [63]. Hence, we assumed that both oculomotor systems are functionally segregated even at basal ganglia level. This hypothesis proved to be correct because in a subgroup of patients engaged in tasks which involved scanning as well as visually guided saccades, we observed that different EM-related neurons were involved in each of the tasks (Table 4). The principle of functional segregation in the control of voluntary and automatic EM had already been previously implied in connection with the interpretation of deep brain stimulation effects [16] or cerebellar lesions [24]. Animal studies have identified spatially segregated functional territories for the control of saccadic EM in the basal ganglia [64], [65]. In primates, the majority of visuo-oculomotor neurons were found in the ventral part of the STN, one third of them being active during reflexive externally triggered saccades and another third being active predominantly during internally triggered (memory guided) saccades [10]. Our results go even further in terms of this specialization hierarchy. Apart from the segregation of populations of EM neurons for scanning movements and visually guided saccades, we identified a higher degree of segregation in all three nuclei neurons. In fact, some neurons responded exclusively to a specific kinematic parameter of the EM associated with an increasing or decreasing firing rate depending on whether or not the eye had reached a particular position, velocity or acceleration of movement (Figure 3).

Some of our results conform to the opposite principle arising from the convergence of cortico-striato-pallido-thalamic projection, i.e. from input nuclei which are larger, to output nuclei which are smaller [23], [66] implying that initially complex information undergoes compression and simplification on its way to the output [67], [68]. Indeed, a small percentage of the STN neurons showed the same neuronal activity in both types of tasks (Table 4). The convergence theory is supported by our observation of 5–8% of STN neurons, whose activity correlated with several kinematic parameters simultaneously (Table 3, 4) suggesting the presence of universal oculomotor neurons. This is in agreement with previous findings of STN neurons which become activated by switching from automatic to voluntary controlled EM [9], with the STN neurons activated from saccades and also during passive movements of the limb [21], with the SNr neurons activated during both pursuit and saccadic EM [13], or with anatomical connections documenting overlap between saccadic and pursuit oculomotor system at the brainstem level [69]. The functional convergence is further supported by the STN deep brain stimulation joint effect on the oculomotor and motor system of the neck and trunk in Parkinson’s disease, marked by simultaneously improved orienting eye-head movements [45] or by improved oculomotor performance associated with body turning [70].

### Time relation between EOG and neuronal activity

In our study, the eye-movement neurons in the STN, SNr or GP were not firing solely in a particular phase of the scanning EM task. In all three nuclei, these neurons became active 200–400 ms before EM, in its course and also 200–400 ms after its onset (Figure 3D). While STN neuronal activity expressed in saccade-related potentials already began 0.8–1.8 s before the saccade, suggesting the involvement of nonspecific readiness non-motor mechanisms [20], single unit neuronal STN and SNr activity culminated within 250 ms after the saccade onset [21] suggesting monitoring or sensory function. Our results are more in agreement with observations of the STN showing modified neuronal activity before, during and after the saccade [10]. This means that scanning EM-related neurons of the STN could be involved in all the preparatory, executive and monitoring phases of EM. This cannot be concluded for GP and SNr due to a relatively low amount of data.

### Limitations

As there were several limitations we should interpret our results with caution. The main problem arised from the impossibility of using infra-red oculography or two-channel EOG during surgery. While their use would definitely have improved the accuracy of the kinematic parameters during EM, they would also have interfered with the established implantation procedure. The use of single-channel EOG, which failed to capture the full extent of free-direction EM and yielded no more than EM projection into a one-dimensional space, is clearly a limitation which to some extent compromised the sensitivity of our study. Another limitation is connected with the assessment of neuronal activity during the oculomotor tasks based on just correlation analysis. Neuronal firing does not have to relate to EM activity alone but it may also reflect visual perception, planning, visuo-spatial attention or other cognitive processing which coincide with oculomotor activity. In addition, our results could be affected by the fact that our data was obtained from patients with Parkinson’s disease in whom abnormal saccadic EM were repeatedly reported [42], [44], [46], [71], [72], [73], [74]. Whether any abnormalities exist in Parkinson’s disease during scanning EM also is not clearly known since, with the exception of one study which showed a deficit in trans-saccadic working memory [75], no-one has systematically focused on scanpath or other parameters of complex exploratory EM in these patients.

## Conclusions

As our results showed, the STN, SNr and GP contain neuronal populations related to scanning EM. Their representation reached about 20% in each of the three nuclei. Basal ganglia are thus not limited to previously described saccade control and perhaps play a more general role in EM circuitry. Oculomotor systems responsible for the execution and monitoring of scanning EM and visually guided saccades are mostly segregated as suggested by neurons involved exclusively in one of two EM tasks or by neurons selectively co-activated in association with a specific kinematic parameter. However, some functional overlap of the two oculomotor systems does exist, albeit confined to small groups of neurons conforming to the complementary convergence principle. Further studies combining clinical and electrophysiological approaches are needed to clarify the role of the basal ganglia in automatic and voluntary oculomotor behavior. We should emphasize, that the large representation of basal ganglia neurons showing activity during all phases of the EM is also an argument for taking them into account when designing new tasks using single unit microrecording. Many visual, ocular or motor experiments are potentially oculomotor in their nature which may compromise results if the EM-related neuronal activity was not considered.

## Supporting Information

Figure S1 **Positions of the eye movement-related neurons along dorso-ventral microelectrode trajectory within the basal ganglia.** A – length of the subthalamic nucleus (STN), B – length of the globus pallidus (GP) and C – length of the substantia nigra pars reticulata (SNr) explored intraoperatively by the five microelectrodes in both the left and right hemispheres and projected to one-dimensional space aligned to the ventral border of the STN and GPi and to the dorsal border of the SNr. Position of each neuron along the dorso-ventral axis is shown in each subject. The proportion of eye movement-related neurons (EM) was significantly higher in the ventral part of the STN.
(TIFF)
