# Supplementary figures and images for: Basal Ganglia Neuronal Activity during Scanning Eye Movements in Parkinson’s Disease
Source: PLoS One. 2013 Nov 6;8(11):e78581. doi: 10.1371/journal.pone.0078581 (PMC3819366; doi:10.1371/journal.pone.0078581)

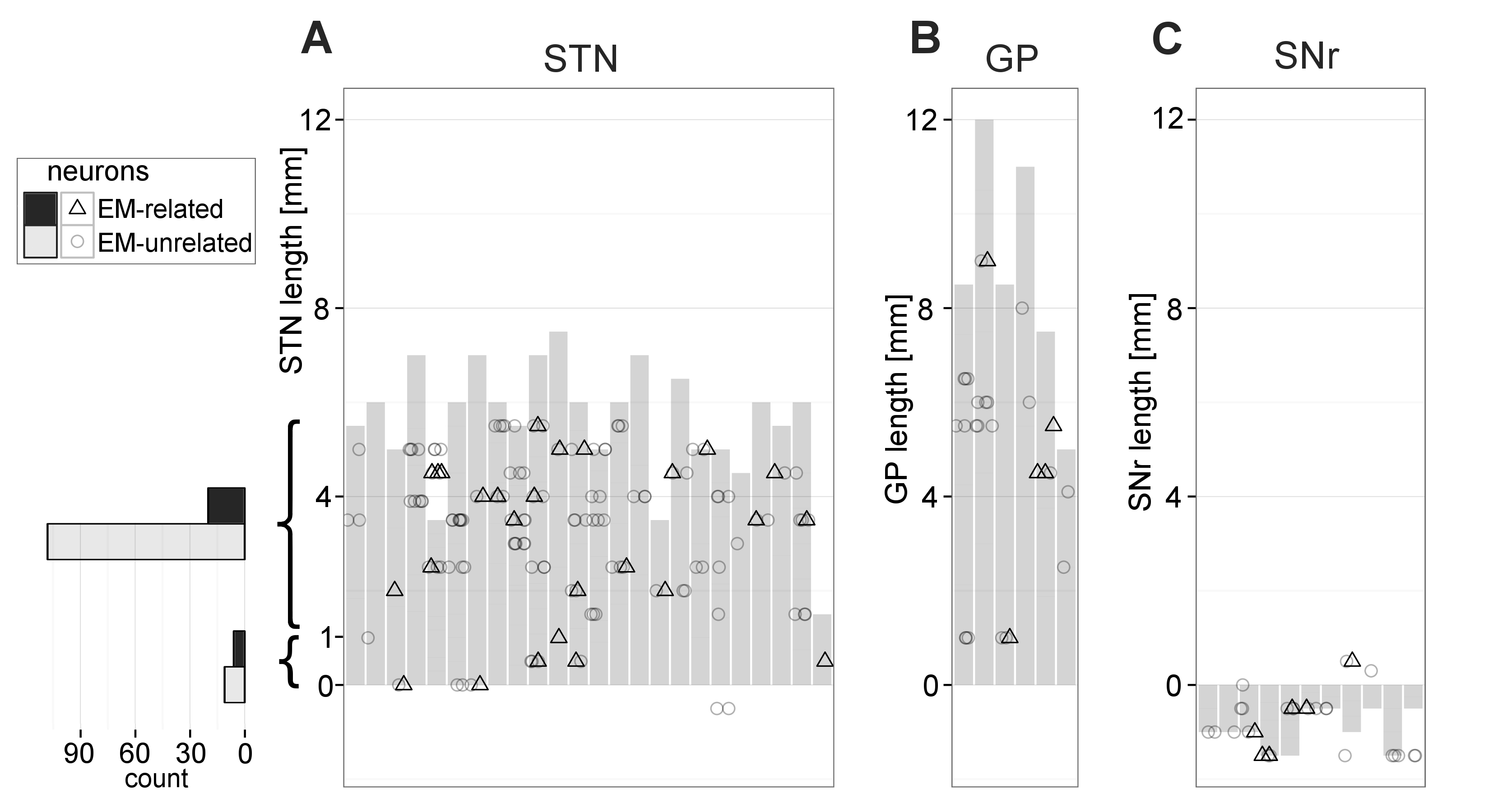

Supplement: Figure S1 — Positions of the eye movement-related neurons along dorso-ventral microelectrode trajectory within the basal ganglia. A – length of the subthalamic nucleus (STN), B – length of the globus pallidus (GP) and C – length of the substantia nigra pars reticulata (SNr) explored intraoperatively by the five microelectrodes in both the left and right hemispheres and projected to one-dimensional space aligned to the ventral border of the STN and GPi and to the dorsal border of the SNr. Position of each neuron along the dorso-ventral axis is shown in each subject. The proportion of eye movement-related neurons (EM) was significantly higher in the ventral part of the STN. (TIFF) [file pone.0078581.s001.tiff]
